# Ulcerative Lichen Planus in Childhood

**DOI:** 10.1155/2013/874895

**Published:** 2013-12-17

**Authors:** Chiyadu Padmini, K. Yellamma Bai, Vinil Chaitanya, M. Shilpa Reddy

**Affiliations:** ^1^Department of Pedodontics and Preventive Dentistry, Malla Reddy Institute of Dental Sciences, Suraram, Zediametla, Hyderabad 500055, Andhra Pradesh, India; ^2^Department of Pedodontics and Preventive Dentistry, Malla Reddy College of Dental Sciences for Women, India; ^3^Department of Oral Medicine and Radiology, Malla Reddy Institute of Dental Sciences, Suraram, Zediametla, Hyderabad 500055, Andhra Pradesh, India; ^4^Department of Conservative Dentistry and Endodontics, Malla Reddy Institute of Dental Sciences, Suraram, Zediametla, Hyderabad 500055, Andhra Pradesh, India

## Abstract

Lichen planus (LP) is a chronic inflammatory mucocutaneous condition which is relatively common in adults but rarely affects children. The present study is a report on an unusual case of ulcerative oral LP involving the dorsum of tongue in a 12-year-old boy. Patient complained of painful oral lesion on the tongue which was burning in nature and obstructing talking and eating spicy foods. On intraoral examination, a white ulcerative lesion on the dorsum of tongue was observed. Diagnosis was made based on clinical examination and histopathological features. We instituted local treatment and patient responded well to the treatment. Although rarely reported in childhood, lichen planus should be considered in a differential diagnosis of hyperkeratotic, reticular, and ulcerative lesions of the oral mucosa in children.

## 1. Introduction

Lichen planus (LP) is an autoimmune, chronic, inflammatory disease that affects mucosal and cutaneous tissues. The exact etiology of LP is unknown, but it is believed to result from an abnormal T cell-mediated immune response in which basal epithelial cells are recognized as foreign because of changes in the antigenicity of their cell surface [1]. Oral lichen planus (OLP) is a common disease in the middle aged and elderly population and has a prevalence of about 0.5% to 2%. By contrast, oral lichen planus in childhood (OLP) is rare and it was first reported in the 1920s. Oral mucosal involvement in adults itself accounts for 0.5% to 19%, while in children, it is very uncommon [2].

The oral lesions are more pleomorphic than those of their cutaneous forms and subtypes are categorized as reticular, papular, plaque-like, atrophic, erosive, and bullous [3]. The erosive form is extremely rare in children and few reports on this subject have been published in the literature.

Herewith, we are presenting a case of a 12-year-old boy having erosive lichen planus without cutaneous involvement, who responded very well to the treatment. This paper also reviews ulcerative oral lichen planus in children and emphasizes its diagnosis from other oral white and red lesions in children.

## 2. Case Report

A 12-year-old boy reported to the Department of Pedodontics and Preventive Dentistry, with the chief complaint of ulcer on his dorsum of the tongue which is causing burning sensation on consuming spicy foods from past 1 year (Figure 5).

**Figure 1:**
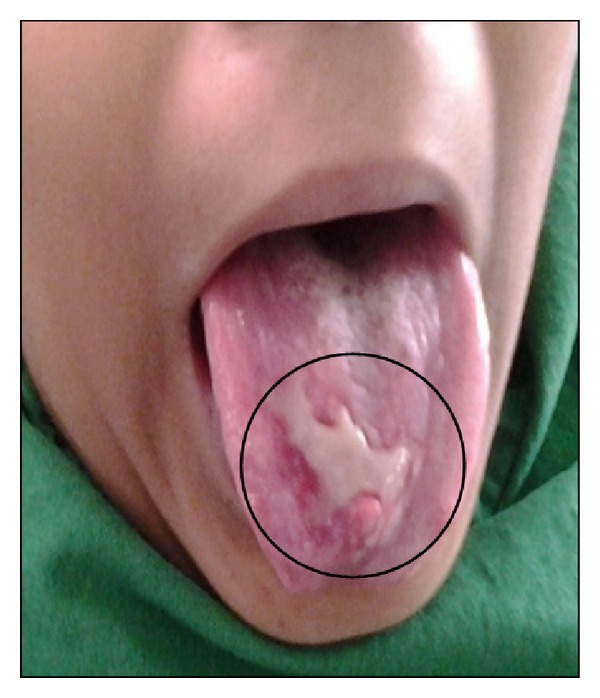
Dorsum of tongue showing ulcerative lesion.

There is no significant medical history observed. On extra oral examination, patient was normal. On intraoral examination, a single irregular red and white ulcerative lesion measuring approximately 2.5 × 1.0 cm in size with granulation tissue at the centre surrounded by an inflammatory red border on the dorsum of the tongue was noticed. There was a depapillation of filiform papillae in and around the lesion (Figure 1). Oral hygiene of the patient was good without any dental restorations.

The differential diagnosis was lichen planus and lichenoid lesions. To exclude lichenoid reaction, we investigated his medical status and there was no history of any drug intake. The patient and his parents also denied any habits that may potentially cause oral mucosal ulcerations.

**Figure 2:**
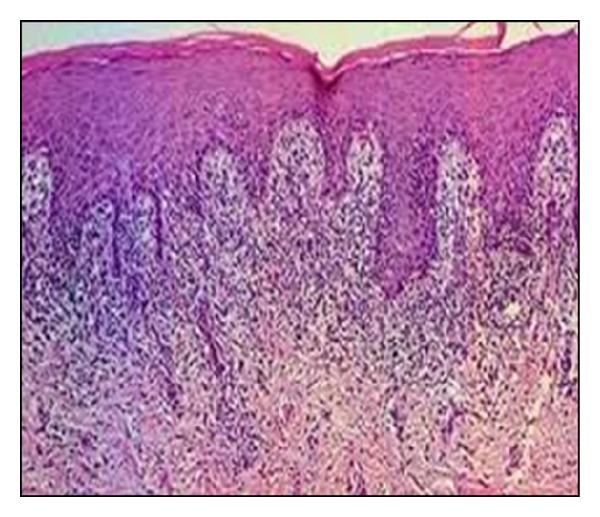
Photomicrograph (5x magnification) of the lesion.

Histopathological examination showed hyperparakeratosis of stratified squamous epithelium and basal cell degeneration with dense band-linked lymphocytic infiltration at the epithelial-connective tissue interface (Figure 2). Both clinical and histopathological features were consistent with ulcerative oral lichen planus.

**Figure 3:**
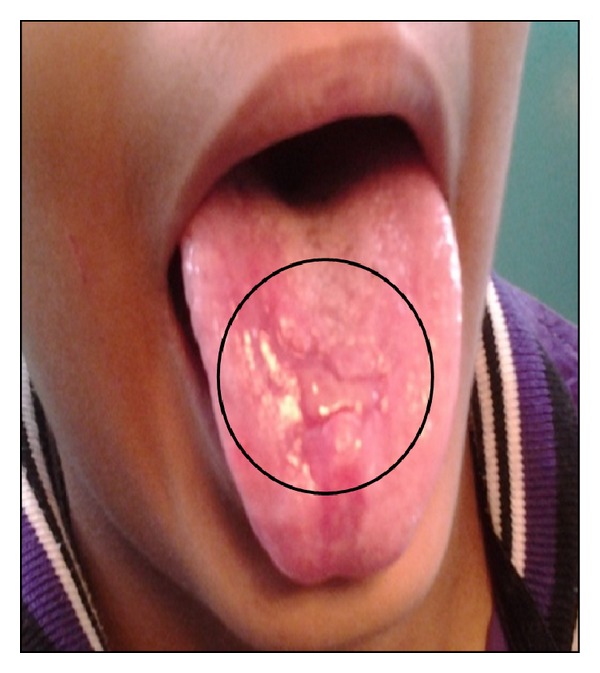
Mid treatment (15th day of treatment) showing reduction in size & healing of the ulceration.

**Figure 4:**
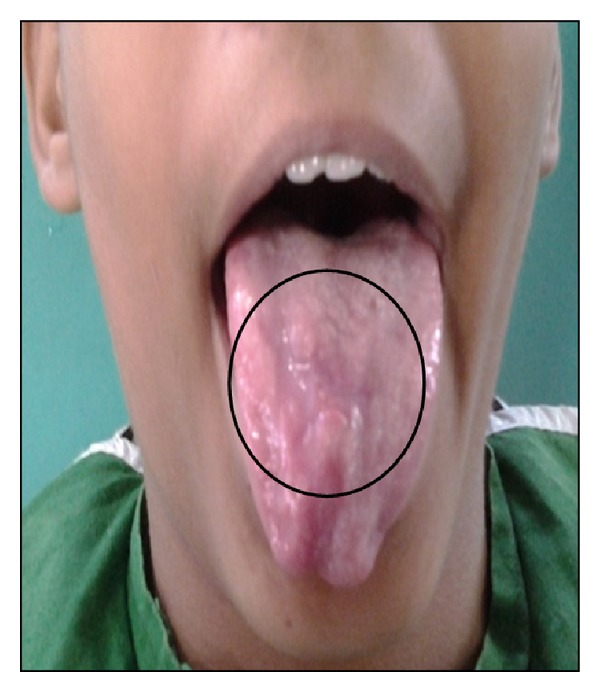
Complete healing of ulcer on tongue (after 30 days of treatment).

**Figure 5:**
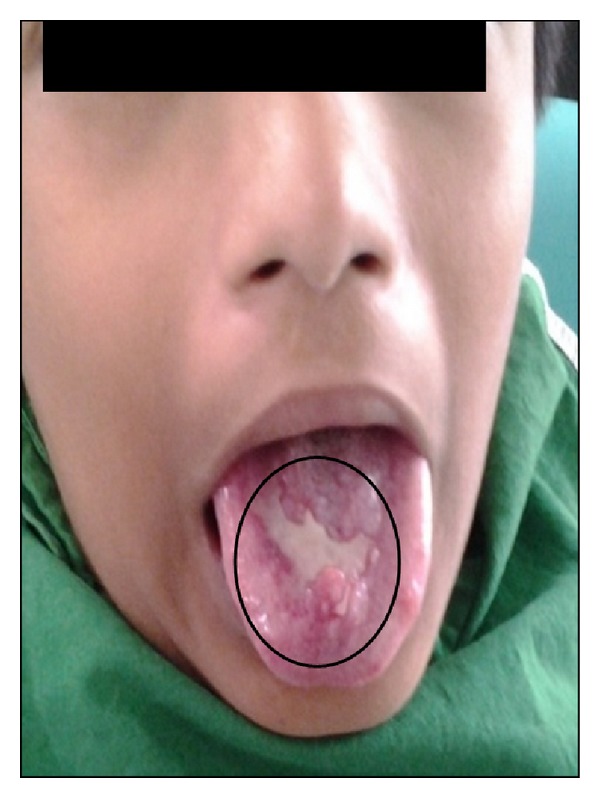
Pretreatment photograph showing ulcer on dorsum of tongue.

**Figure 6:**
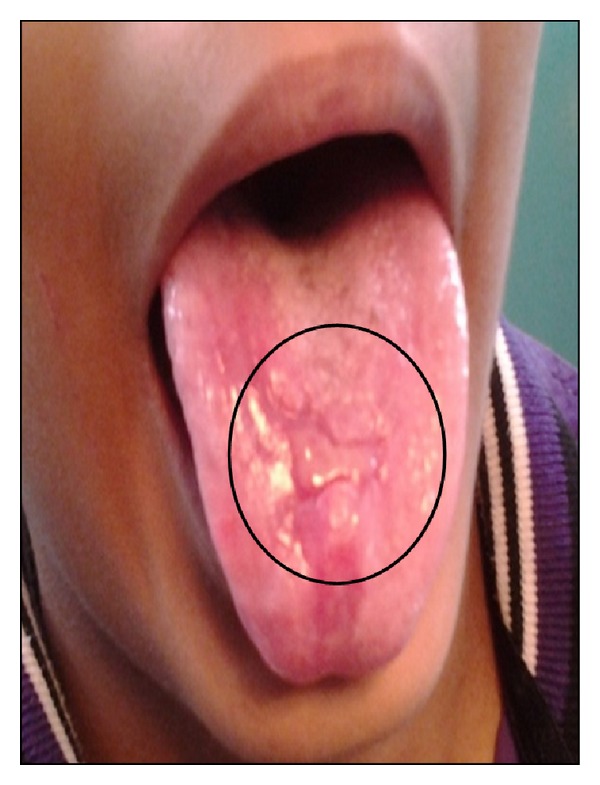
Mid treatment photograph showing resolution size of the lesion.

**Figure 7:**
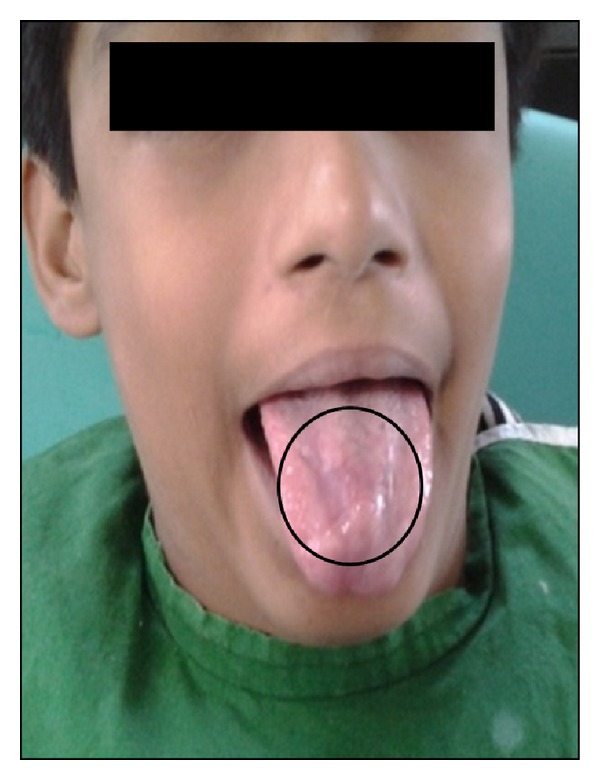
Posttreatment photograph showing complete healing of ulceration.

Specific treatment for ulcerative OLP was topical 0.1% triamcinolone acetonide combined with 1% clotrimazole 3–5 times per day for a duration of one week. Topical anesthetic was given for the pain relief. First review of the patient after 15 days showed significant reduction in both symptoms and signs of the oral lesions (Figure 3). After 15 days, there was good prognosis in the recovery of ulcerative lichen planus (Figure 6). Erosive oral ulcerative oral lichen planus had completely healed at the end of 30 days (Figures 4 and 7). Patient was observed on periodic recall followup.

## 3. Discussion

Oral lichen planus in childhood (OLPc) is rare and only a few reports are available in the literature [4]. Oral lichen planus can be divided into a hyperkeratotic (white) variant, commonly without symptoms, a reticular type with Wickham striae (often symmetrical), and papular and plaque-like types.

The atrophic/erythematous (red) variant and the erosive/ulcerative (yellow) variant often have persistent symptoms of pain or stinging aggravated during talking and eating spicy foods. These variants may occur together in one patient or may transform from one to another. The lesions were found more commonly on the buccal mucosa (often symmetrical), lateral margins of the tongue, gingiva, and lips.

Whereas cutaneous LP is self-limiting, ulcerative OLP is chronic, and rarely undergoes spontaneous remission. The family history of LP is more commonly positive in patients with LP in childhood than in adulthood. The exact cause of ulcerative OLP remains unknown, but an immune-mediated (T cell dependant) pathogenesis has been reported.

OLP in childhood was first described in 1920 and since then only few articles have been published and most of the studies have suggested that childhood LP is more common in tropical countries like India [5]. Sharma and Maheshwari reported 50 children with LP and with concomitant oral lesions in 15 of them and they stated that the oral mucosa seems to be less commonly involved in children with LP than in adults [6].

Predisposing conditions such as graft-versus-host disease, active hepatitis, and hepatitis B immunization are rather frequently mentioned in these reports. Kanwar and Kumar reported only one case having oral ulcerative lichen planus out of 25 patients with cutaneous lichen planus [7].

The mean interval between vaccination and LP onset was three years, ranging between three months and 11 years. Handa and Sahoo reported 87 patients with childhood LP in India. Seven patients showed involvement of the oral mucosa and only one patient had oral ulcerative lichen planus without skin involvement [8].

A 10-year retrospective study was done by Ronald Laeijendecker et al., which was comprised of 10,000 patients below 18 years, with a boy to girl ratio of 1 : 1, and which has shown that the only 3 patients (0.03%) were diagnosed with oral lichen palnus [9]. A study done in the United Kingdom by Alam and Hamburger on boys aged between 6 and 14 years over a period of 20 years has proved that only 6 boys have been diagnosed with OLP and interestingly among 6 patients, 4 were Asians [10]. In 1994, Scully et al. reported 3 girls with OLP, one of whom was from Asian origin [11].

The difference in the prevalence of OLP in children (0.03%) versus that of OLP in adults (0.5%–2%) is understood by less number of associated systemic diseases in children, autoimmune diseases, infections, drug usage, and dental restorations in childhood; this may reduce the risk for developing OLP in childhood [12]. Moreover, the diagnosis of OLP may be missed due to irregular dental checkups, lack of symptoms, and ignorance by clinicians in diagnosing the condition.

The prognosis and the effect of treatment in OLP in children seem to be more favorable than in OLP in adults, which usually persists for many years in spite of intensive treatment and thorough investigation of associated factors. Malignant transformation of ulcerative OLP in adults is 0.07% to 5%; however, malignant transformation of OLP in children is not documented in the literature till now [13].

## 4. Conclusion

Oral lichen planus in childhood is rare, especially erosive form; diagnosis should be based on children presenting with ulcerative white lesion in oral cavity. The schedule of followup of OLP in children should be 7 days, 15 days, and 30 days after diagnosis to assess healing. Patient should be reviewed twice a year for regular followups after complete progress of the present condition. However, generally, the prognosis of oral lichen planus in childhood seems to be more favorable compared to adults.
